# Effect of electrospinning process variables on the size of polymer fibers and bead-on-string structures established with a 2^3^ factorial design

**DOI:** 10.3762/bjnano.9.231

**Published:** 2018-09-17

**Authors:** Paulina Korycka, Adam Mirek, Katarzyna Kramek-Romanowska, Marcin Grzeczkowicz, Dorota Lewińska

**Affiliations:** 1 Nalecz Institute of Biocybernetics and Biomedical Engineering, Polish Academy of Sciences, Warsaw, Poland

**Keywords:** bead-on-string structure, electrospinning, factorial design, polymer fiber

## Abstract

This work examines the effect of selected process parameters on the diameter of uniform and heterogeneous fibers (with and without bead-on-string structures) and the size of beads obtained during the electrospinning process. A 2^3^ factorial design was performed to determine the influence of the following factors: electrical voltage, flow rate and dynamic viscosity of the poly(vinylpyrrolidone) ethanolic solution. Factorial design enables the analysis of the mathematical relationship between the chosen factors and the response with a minimum number of experiments. The factor having the most significant impact on the size of beaded fibers and beads was the solution viscosity, while the voltage had the greatest influence on the bead-free fiber diameter. The interactions between the studied factors were also analyzed. It was found that the presented method can be used for the design of an optimal and cost-effective electrospinning process, allowing the desired product to be obtained with expected features.

## Introduction

Since the beginning of the 21st century, the interest in electrospinning processes has been growing constantly. The reason for this is the wide range of diameters possible (i.e., from nanometers to several micrometers) for polymer fibers using this electrostatic method [1–2]. In addition, the technique is rather easy to employ and cost-efficient [3–4]. Currently, by modifying the experimental setup and controlling the properties of the polymer solutions, it is possible to obtain fibers of different structure: porous, smooth, core–shell, hollow structures and layer-by-layer stacked films or uniaxially aligned arrays [2]. Because of the variety of obtained structures that are possible, electrospun nanofibers find applications in well-established technologies, as well as in new fields of scientific and industrial interest. They are considered to be potentially useful in areas such as nanoelectronics, medicine (e.g., wound healing, tumor therapy, inhalation therapy), filtration processes, textile manufacture, nanocatalysis production, etc. [5–6].

Current research on the electrospinning method is concentrated on the conditions of the process [7–8], fiber characteristics and their potential application. As of today, there are several studies regarding the influence of process conditions as they relate to the properties of the obtained product [3,5,8–10]. Not only do they concern the values but also the change of process parameters such as: polymer solution properties (type of polymer, type of solvent, solution viscosity [11], surface tension, conductivity [11–12], etc.) and process variables (electrical voltage delivered to the nozzle tip [13], distance between the nozzle tip and the collector, solution flow rate [13], humidity [9,14–15], temperature [15], shape and type of the collector [5], etc.). What should be emphasized is that electrospinning is a complex process with a considerable number of factors that may influence the final product properties. Hence, establishing a complete description of all the occurring phenomena poses a real challenge.

One of the most interesting works in this area is the one by Yuya et al. [10], which discusses the impact of abovementioned process parameters on the obtained nanofiber size and structure. According to this study, the higher the concentration of polyvinylpyrrolidone (PVP) ethanolic solution (higher solution viscosity) used, the thicker the fibers. In addition, both the type of solvent (its physicochemical properties) and the water content in the solvent turned out to be influential in terms of the fiber surface morphology. It was also concluded that methanol and ethanol were best suited for electrospinning of PVP, in contrast to water and dimethylformamide (DMF), which prevented the polymer from spinning. What is more, the higher the water content of the solvent, the less uniform fibrous mats were obtained. Finally, the effect of humidity was also examined: the higher the humidity, the less homogeneous mats were obtained (during electrospinning the solvent evaporates completely and the fibers become glued together).

Another interesting work on the impact of process conditions on the surface morphology of nanofibers is the one by Deitzel et al. [7]. This work focuses on the influence of two process variables: voltage and concentration of the polymer solution of polyethylene oxide (PEO) dissolved in water. In this study it was observed that the increase in electrical voltage changed the shape of the electrospinning jet. In addition, it was shown that the voltage was strongly correlated with the formation of beads, which were recognized by authors as defects. An increase in electrical voltage caused an increase in the density of beads in the obtained polymer mats. It was also concluded that the properties of the polymer solution (concentration, viscosity and surface tension) had the biggest influence on the size of fibers obtained in the electrospinning process. In terms of solution concentration, the proportional relationship between the polymer concentration and the fiber size was noticed. Besides, for concentrations higher than 8%, a bimodal fiber size distribution was obtained.

Many authors describe the formation of bead-on-string structures during the process of electrospinning as an undesirable phenomenon. They clearly state that there is a certain limit of the polymer solution concentration below which the electrospun fibers have characteristic beads in their structure [16–18]. As the concentration of the solution increases, the size of the beads increases too, the average distance between them is greater, their shape changes from spherical to spindle-like and the fiber diameter increases at the same time [17]. Although such structures in the fibrous mats are generally treated as defects that should be avoided [7,18], there are some studies in which their promising applications are outlined. In this context, fibrous mats with beads can be used among others as: coatings with hydrophobic properties [19], membranes for fog harvesting [20], drug delivery systems with encapsulated therapeutic substances [21]. Therefore, it is fully justified to dedicate a study to the analysis of conditions in which the beads can be optimally obtained.

Although a significant number of studies on the influence of various parameters on the electrospinning process have been carried out so far, the selection of optimal conditions is still a serious challenge. One of the methods that may enable this problem to be solved is the concept of factorial design, which allows the effects of the selected factors to be understood and/or to model the relationship between the output and input variables with a minimum number of experiments [22]. This method is widely used in various technological problems (e.g., spray drying process [22–24] or genetic algorithms [25]), as it allows complex systems to be analyzed and to identify the most important information about the impact of factors in a discussed case. However, to the authors’ knowledge, factorial design is not a common tool for the analysis of the electrospinning process or for the morphology of its products (fibers and beads). There are only a few research articles showing the application of this method. Coles et al. examined the influence of various process parameters on polylactic acid and poly(vinyl alcohol) electrospun fibers [26]. Nottelet et al. used factorial design to optimize the manufacture of small diameter vascular grafts made of poly(ε-caprolactone) electrospun nanofibers [27]. However, works on the implementation of the factorial design to describe bead-on-string structures have not been conducted yet. All of the dependencies, described in the abovementioned studies, are usually established empirically and by the qualitative description of the obtained fibrous mat structures [14] and thus in a subjective way. The mathematical description of the factorial design allows the problem to be solved in a more objective manner.

The factorial design, a tool used in this work, can be useful for designing the fibers obtained during the electrospinning process. The literature analysis indicates that there are three process parameters with the most impact on the structure of fibrous mats: polymer solution dynamic viscosity (μ), electrical voltage (*U*) and solution flow rate (*Q*). The aim of the present study was to assess the influence of these factors on the fiber diameter and bead size.

## Experimental

### Materials

Polyvinylpyrrolidone (PVP, 1300 kDa), purchased from Sigma-Aldrich, was chosen because it can be prepared with harmless and easily accessible solvents such as ethanol. The solutions of PVP dissolved in ethanol 96% (w/w), purchased from Polmos (Poland), were made with the following concentrations: 8% (w/w), 12% (w/w), 14% (w/w), and 20% (w/w).

### Electrospinning setup

**Figure 1:**
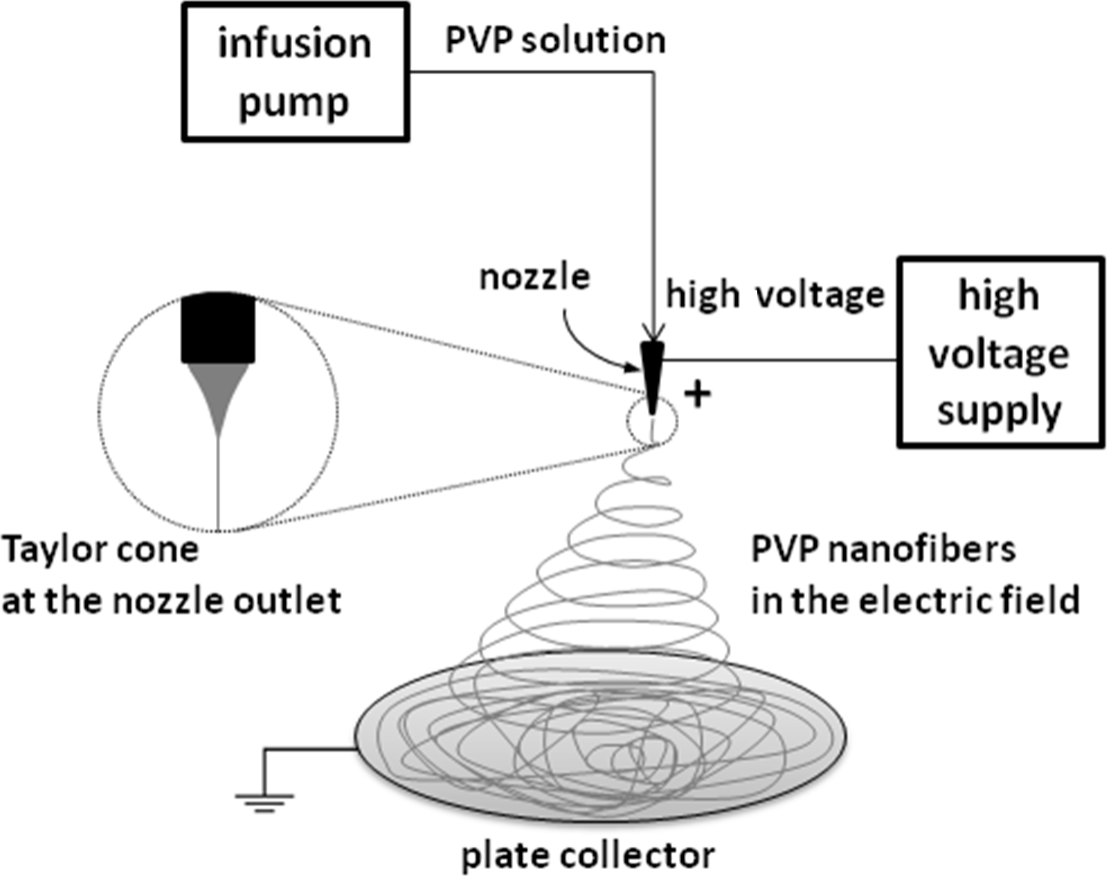
Scheme of the apparatus for the electrospinning process.

The weighted portion of PVP solution was delivered to a steel nozzle with an infusion pump. The nozzle had an inner diameter of 0.6 mm (Figure 1). A round aluminum collector (thickness: 0.12 mm; diameter: 40 mm) was located about 15 cm below the metal nozzle. The high voltage was set within the limits of 7.5–15 kV and the flow rate of the solution in the range of 0.6–1.2 mL/h. The collection time of the fiber was adjusted to collect the same polymer solution volume in each study (0.06 mL of solution was used to make one sample). The experiments were carried out at room temperature (about 25 °C), the humidity did not exceed 40%. The diagram of the self-made electrospinning setup is shown in Figure 1.

### Characterization procedures

**Figure 2:**
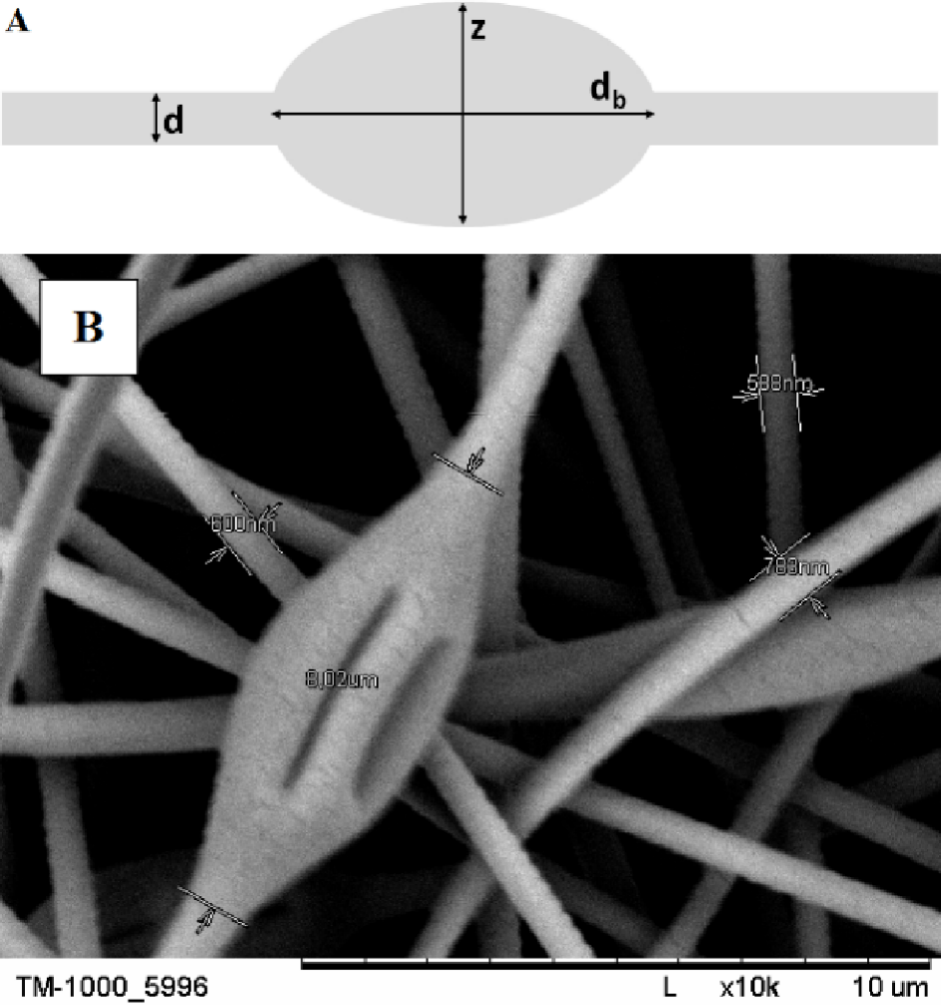
(A) A schematic of the measuring procedure of the size of beads and the diameter of the fiber (where *z* ≥ 3*d*). (B) An exemplary SEM micrograph of the obtained fibers and beads showing the measurement procedure (conditions: PVP 12%; 0.6 mL/h; 7.5 kV).

The dynamic viscosity of solutions was measured with a RheolabQC rotation viscometer at a predetermined temperature (25 °C). A scanning electron microscope (SEM, Hitachi TM-1000) was used to analyze the structure of the obtained electrospun mats. The samples of electrospun mats were sprayed with a 15 nm thick gold layer. Using the computer software provided with the microscope, the diameters of 20 randomly selected fibers or beads, obtained from each PVP solution, were measured (Figure 2B). The methodology described above is shown schematically in Figure 2A. It was assumed that the width of the bead (*z*) must be at least three times greater than the diameter of the fiber (*d*) on which the bead is located. The length measured between the places where the fiber diameter begins to change was taken as the size of the bead (*d*_b_). The geometric shape of the liquid meniscus at the nozzle tip was recorded using a monochromatic industrial camera (Mintron MTV-1361CA). VirtualDubMod software was used to take pictures of the nozzle outlet. Microsoft Excel software was used to perform the factorial analysis and the 3D graphs were made using the software Origin.

### Factorial design

The impact of the selected parameters on the formation of fibers/beads during the electrospinning process was examined using the factorial design. The factorial design method allows the direct influence of process factors to be determined and possible effects of their interaction.

In this method, the experimental domain must be specified to set up a two-level factorial design. A high (+) and low level (−) is assigned to each factor. The complete model system contains all possible combinations of settings of extreme experimental factors. The model with *k* factors consists of 2*^k^* experimental runs.

For the case with three experimental factors, the response surface model is as follows:

[1]
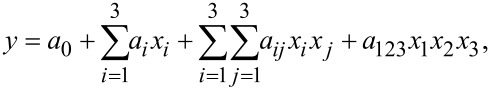


where *y* – the response; *a*_0_*, a_i_*, *a_ij_*, and *a*_123_ – the coefficients; *x*_1_, *x*_2_, *x*_3_, *x_i_*, and *x_j_* – the experimental factors. The constant term *a*_0_ corresponds to the response value when all parameters are at the center point at an average level (*x*_1_ = *x*_2_ = *x*_3_ = 0).

Then the coefficients (*a*) are determined by the encoded values of factors. It is assumed that the high factor level is +1, and the low factor level is −1. After the transformation of the factors, the experimental system takes the form of a matrix, which in the case of three factors is as follows:

[2]
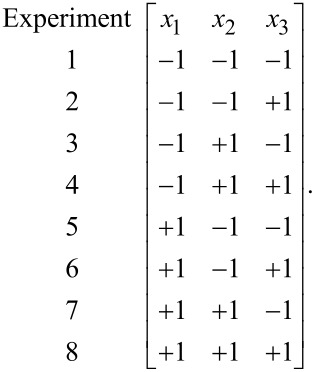


In order to calculate the coefficients *a_i_*–*a_ijk_*, an **X**-matrix is built by extending it by column *I* for a constant expression, and the columns for all possible factor interactions in the model as:

[3]
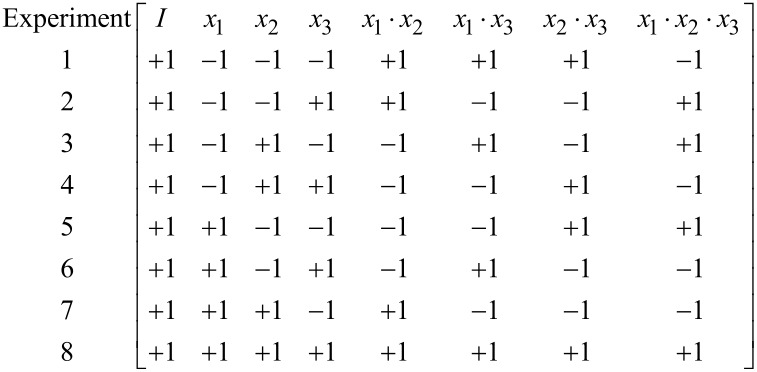


Consequently, the discussed experimental series can be summarized by means of the matrix relation:

[4]
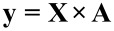


which in the described case corresponds to:

[5]
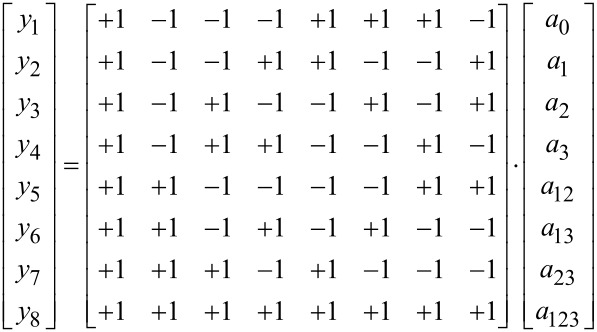


Finally, the coefficients are determined by solving Equation 6 using the least squares method:

[6]
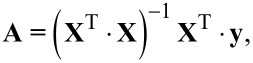


where **A** – set of the coefficients; **X**^T^ – transposed matrix; **y** – response.

The absolute value of a given coefficient *a* determines the influence of the analyzed factor of the model on the response (the higher the coefficient value, the stronger the relationship between the given factor and the response). The sign of the coefficient indicates the character of the dependence: a positive coefficient indicates that the value of the response increases with the increase of the factor value, and a negative coefficient indicates that the relationship is inversely proportional.

### Selection of process factors for the factorial design

The dynamic viscosity values of the polymer solutions used in the investigation are listed in Table 1.

**Table 1:** Dynamic viscosity of polyvinylpyrrolidone (PVP) solutions.

Concentration *C* [%]	Dynamic viscosity µ [Pa·s] (25 °C)

8	0.043
12	0.107
14	0.232
18	0.398
20	0.616

In order to perform a full analysis, it was crucial to select experimental variants carried out under extreme conditions. The high (maximum) and low (minimum) levels of the considered factors (µ, *U*, *Q*) were assigned the values of +1 and −1 accordingly. The particular parameters for the analysis of their impact on the bead-free fiber diameter are shown in Table 2.

**Table 2:** Process variables selected for the factorial analysis for fiber diameter.

Parameter	Low level (−1)	High level (+1)

µ	0.232 Pa·s	0.616 Pa·s
*Q*	0.6 mL/h	1.2 mL/h
*U*	7.5 kV	15.0 kV

The particular factors for the analysis of their influence on the beaded fiber diameter and bead size are shown in Table 3.

**Table 3:** Process variables selected for the factorial analysis of bead size and fiber diameter in the fibrous mats with bead-on-string structure.

Parameter	Low level (–1)	High level (+1)

µ	0.043 Pa·s	0.107 Pa·s
*Q*	0.6 mL/h	1.2 mL/h
*U*	7.5 kV	15.0 kV

**Figure 3:**
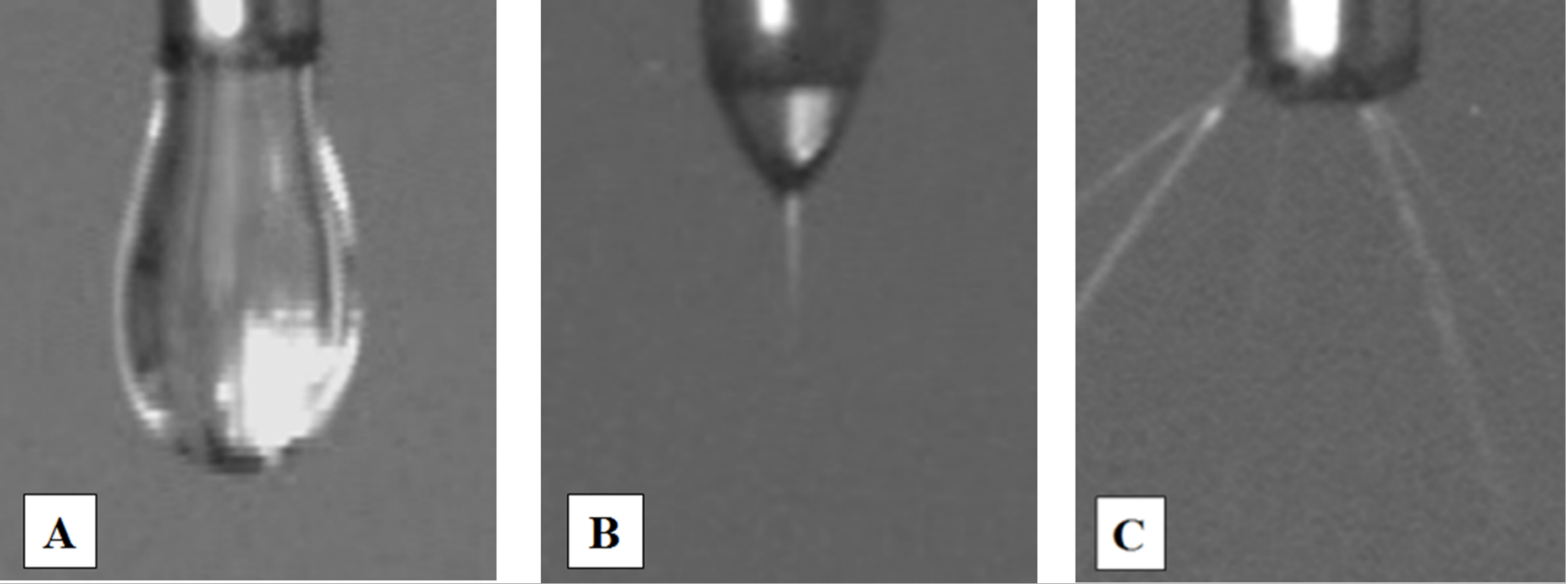
Geometric shape of the liquid meniscus at the outlet of the nozzle for 20% PVP solution with flow rate 1.2 mL/h and electrical voltage: (A) below the low level (4 kV); (B) within the experimental domain (7.5 kV); (C) greater than the high level (17 kV).

The high level of the solution dynamic viscosity is the viscosity at a PVP concentration of 20%, whereas the low level is found at the viscosity where the PVP concentration is a 14% solution under the same conditions. Concentrations above 20% (and thus higher viscosity) induce rapid solvent evaporation and obstruction of the nozzle. Concentrations below 14% (and thus lower viscosity) lead to beaded fibers produced during the electrospinning process. The selection of extreme concentrations was done according to experimental results. The high level value for the solution flow rate is the limit for a stable process and higher values induce droplet formation. Flow rate values lower than the assumed low level are too small to start the electrospinning process. Finally, low and high levels of the electrical voltage are the minimum and the maximum values between which the process is stable, respectively. Below the low level of the voltage, the droplet formation occurs and at values greater than the high level, the voltage splits the solution into several streams. The geometric shapes of the polymer solution meniscus at the outlet of the nozzle under different electrical conditions are presented in Figure 3.

## Results and Discussion

A factorial analysis was carried out for the three cases. The influence of the three factors (voltage, flow rate and viscosity of the PVP solution) on the fiber diameter (without and with beads) and the size of the beads themselves were investigated.

### Structure of electrospun mats

**Figure 4:**
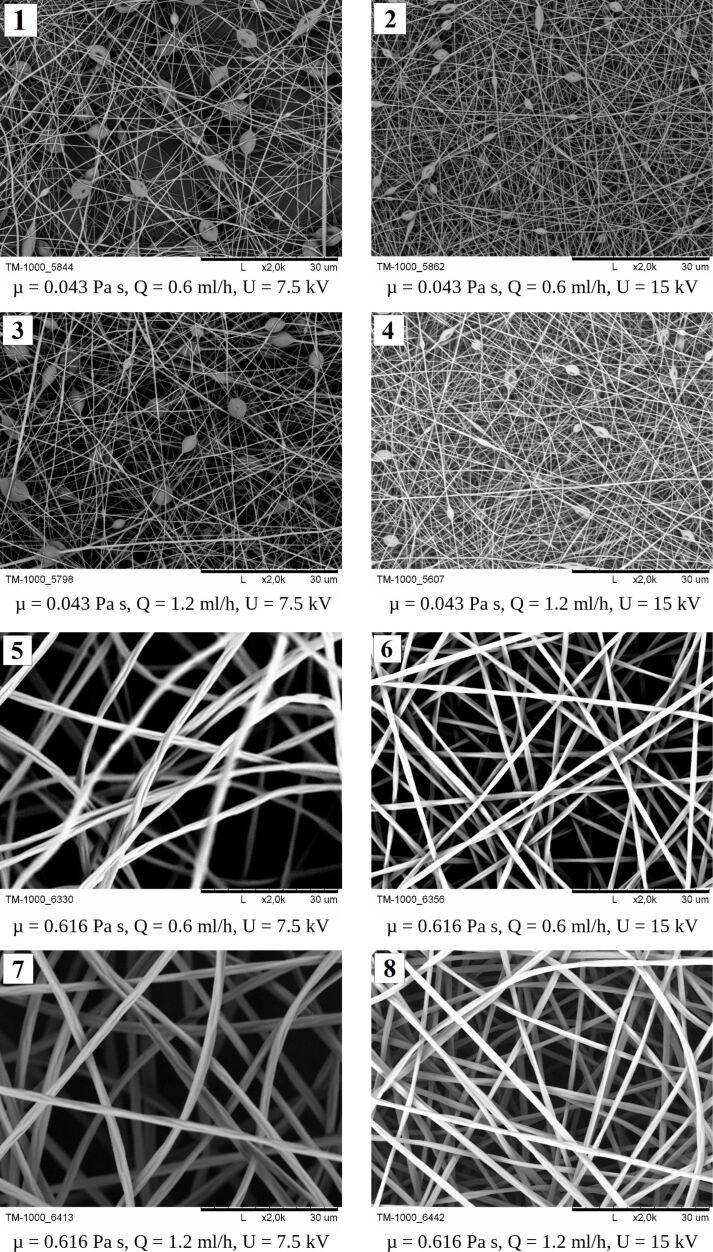
SEM micrographs of nanofibers obtained under various conditions: (1) PVP 8% (µ−, *Q*−, *U*−); (2) PVP 8% (µ−, *Q*−, *U*+); (3) PVP 8% (µ−, *Q*+, *U*−); (4) PVP 8% (µ−, *Q*+, *U*+); (5) PVP 20% (µ+, *Q*−, *U*−); (6) PVP 20% (µ+, *Q*−, *U*+); (7) PVP 20% (µ+, *Q*+, *U*−); (8) PVP 20% (µ+, *Q*+, *U*+), where “+” indicates the high level of the given factor and “−“ the low level of the given factor. The black scale bar underneath all images is 30 μm.

Figure 4 (1–8) shows exemplary micrographs of both beaded and bead-free polymer mats.

According to the pictures of the obtained fibrous mats (Figure 4 (1–4)), it can be noticed that for solutions with low viscosity (below 0.123 Pa·s) structures called beads appear. The increase in viscosity causes the disappearance of these elements in the structure of the mat. The fibers are characterized by a smooth, nonporous surface and their relative arrangement to each other is random.

### Effect on the diameter of the bead-free and beaded fibers and the size of the beads

#### Bead-free fibers

As the result of the experiments, the average bead-free fiber diameter (*D*) was considered. The variants of the experiments and obtained results are presented in Table 4.

**Table 4:** Experimental variants for a 2^3^ factorial design including the average diameter of the obtained beadless fibers (*D*). SD is the standard deviation.

No.	*C* [%]	µ [Pa·s]	*Q* [mL/h]	*U* [kV]	*D* [μm]	SD [μm]

1	14	0.232	0.6	7.5	1.803	0.294
2	14	0.232	0.6	15.0	1.427	0.280
3	14	0.232	1.2	7.5	1.661	0.382
4	14	0.232	1.2	15.0	1.293	0.324
5	20	0.616	0.6	7.5	2.012	0.513
6	20	0.616	0.6	15.0	1.299	0.161
7	20	0.616	1.2	7.5	2.131	0.314
8	20	0.616	1.2	15.0	1.526	0.199

Based on the results presented in Table 4, the influence of the individual process factors (µ, *Q, U*) on the fiber diameter was determined according to the scheme outlined previously. The response surface model, in the form of Equation 7, was obtained.

[7]
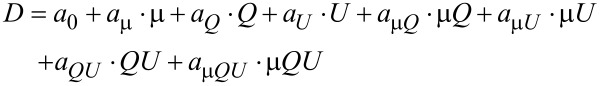


where: *a_i_*–*a_ijk_* – the model coefficients; μ, *Q, U* – the process factors (dynamic viscosity, flow rate, electrical voltage), *D* – the response (average fiber diameter).

In order to determine the model coefficients, a design matrix was prepared assigning +1 values to the high levels of process factors and −1 values to low ones (Table 5).

**Table 5:** Design matrix of a 2^3^ factorial design.

No.	µ [Pa·s]	*Q* [mL/h]	*U* [kV]

1	0.232 (−1)	0.6 (−1)	7.5 (−1)
2	0.232 (−1)	0.6 (−1)	15.0 (+1)
3	0.232 (−1)	1.2 (+1)	7.5 (−1)
4	0.232 (−1)	1.2 (+1)	15.0 (+1)
5	0.616 (+1)	0.6 (−1)	7.5 (−1)
6	0.616 (+1)	0.6 (−1)	15.0 (+1)
7	0.616 (+1)	1.2 (+1)	7.5 (−1)
8	0.616 (+1)	1.2 (+1)	15.0 (+1)

Thereafter, the model matrix **X** (Table 6) was constructed by adding a column *I* to the design matrix corresponding to the constant term *a_0_* in Equation 7.

**Table 6:** Model matrix of a 2^3^ factorial design.

No.	*I*	µ	*Q*	*U*	µ*Q*	µ*U*	*QU*	µ*QU*

1	+1	−1	−1	−1	+1	+1	+1	−1
2	+1	−1	−1	+1	+1	−1	−1	+1
3	+1	−1	+1	−1	−1	+1	−1	+1
4	+1	−1	+1	+1	−1	−1	+1	−1
5	+1	+1	−1	−1	−1	−1	+1	+1
6	+1	+1	−1	+1	−1	+1	−1	−1
7	+1	+1	+1	−1	+1	−1	−1	−1
8	+1	+1	+1	+1	+1	+1	+1	+1

Finally, the values of the parameters *a*_0_–*a*_µ_*_QU_* were determined according to Equation 6. The results are listed in Table 7.

**Table 7:** The model coefficients determining the impact of solution dynamic viscosity (µ), solution flow rate (*Q*) and electrical voltage (*U*) on the bead-free fiber average diameter (*D*).

*a*_0_	*a*_μ_	*a_Q_*	*a*_μ_	*a*_μ_*_Q_*	*a*_μ_*_U_*	*a_QU_*	*a*_μ_*_QU_*

1.644	0.098	0.009	−0.258	0.078	−0.072	0.015	0.013

The corresponding model equation is presented below:

[8]
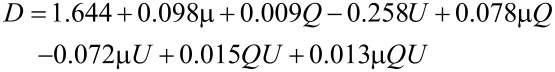


On the basis of the model coefficients presented in Table 6, it can be apparently seen that the electrical voltage (*U*) is the parameter with the greatest influence on the average size of bead-free fibers. Since the coefficient *a*_μ_ is negative, this relationship is inversely proportional. In addition, the diameter of the fiber increases with increasing viscosity of the polymer solution (*μ*), which is the second most influencing factor. The value of coefficient *a_Q_*, is an order of magnitude (or two) smaller than the value of the coefficients *a*_µ_, *a_U_*, thus the influence of the flow rate (*Q*) on the fiber diameter is much smaller than the impact of other parameters.

**Figure 5:**
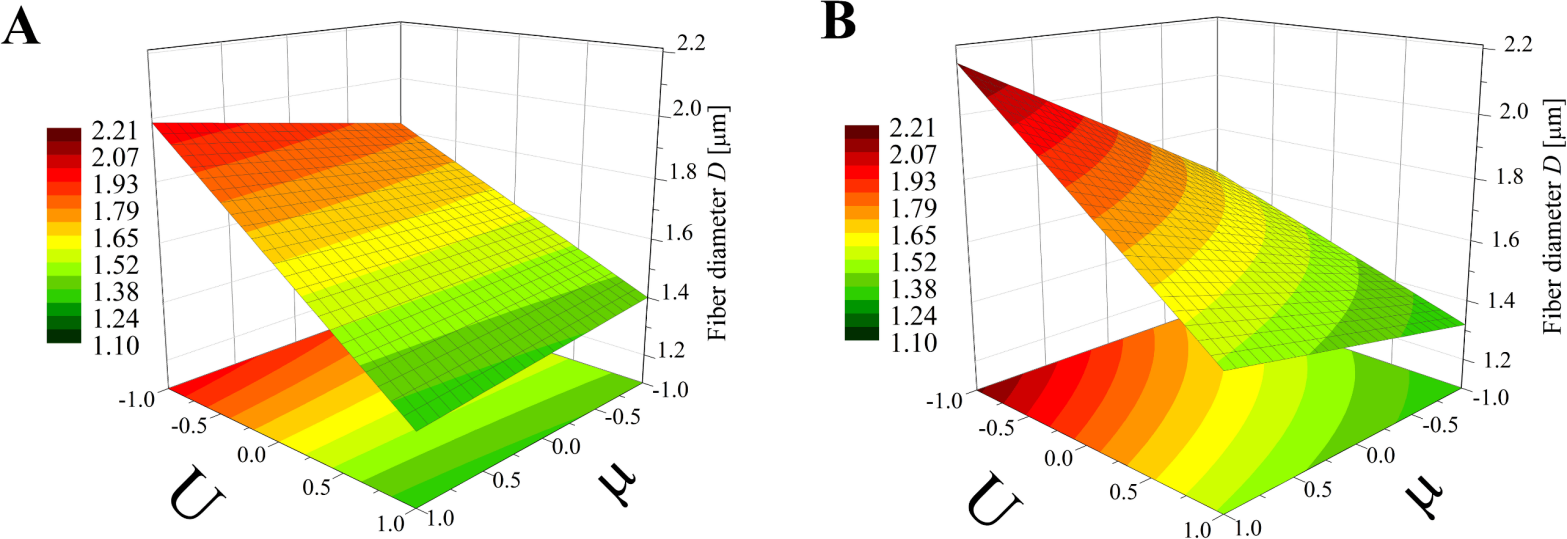
Response surface plots presenting the dependence of the diameter of the obtained bead-free fibers (*D*) on the electrical voltage (*U*) and the dynamic viscosity (µ) of the polymer solution for the (A) minimum flow rate (*Q* = −1) and (B) maximum flow rate (*Q* = 1)*.*

**Figure 6:**
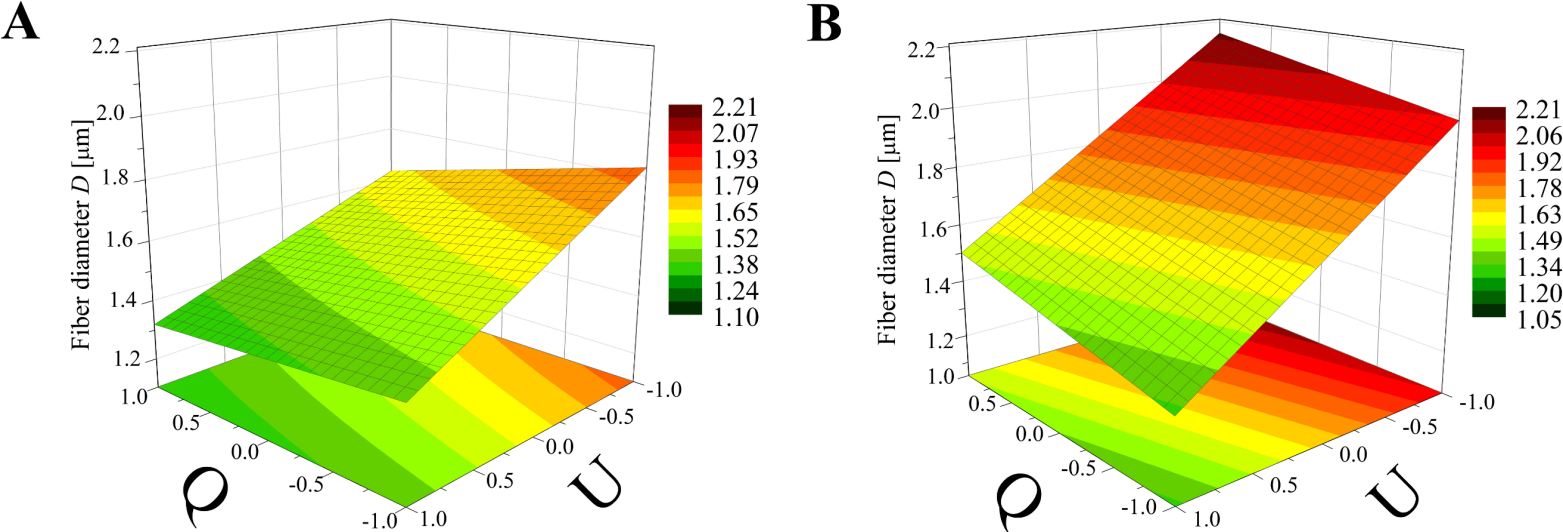
Response surface plots presenting the dependence of the diameter of the obtained bead-free fibers (*D*) on the electrical voltage (*U*) and the flow rate of the polymer solution (*Q*) for the (A) minimum dynamic viscosity (µ = −1) and (B) maximum dynamic viscosity (µ = +1)*.*

**Figure 7:**
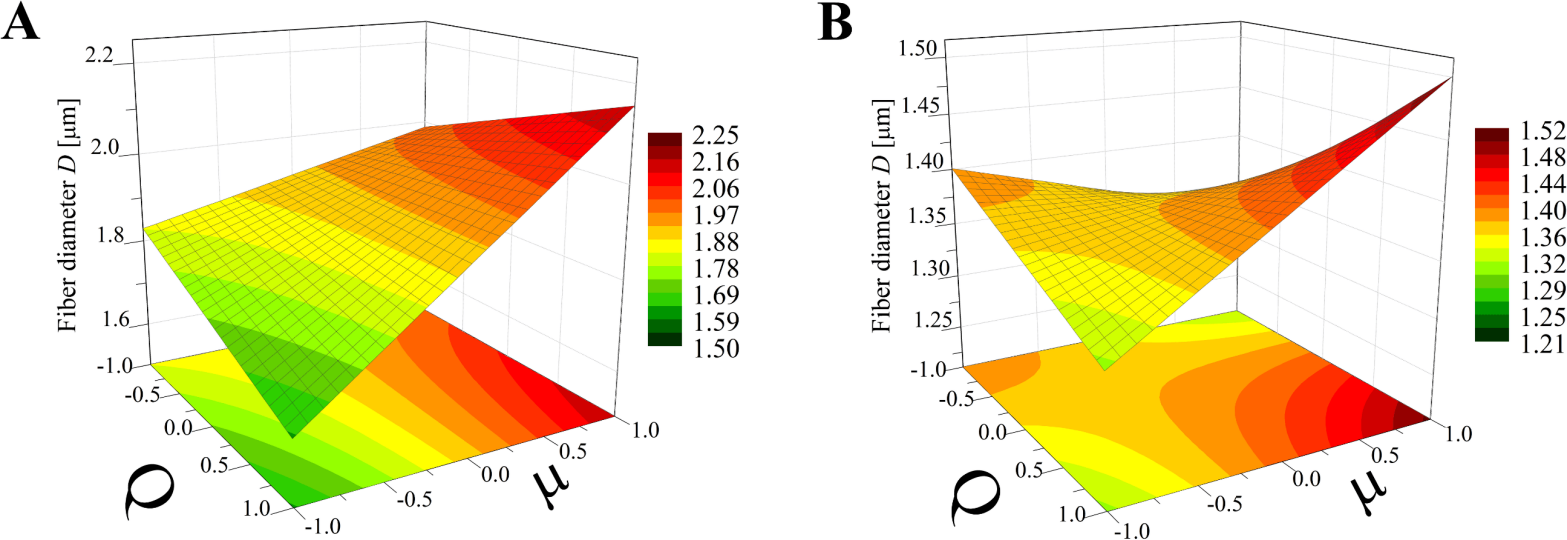
Response surface plots presenting the dependence of the diameter of the obtained bead-free fibers (*D*) on the dynamic viscosity (µ) and the flow rate (*Q*) of the polymer solution for the (A) minimum electrical voltage (*U* = −1) and (B) maximum electrical voltage (*U* = +1).

The obtained model (Equation 8) was used to create three-dimensional plots, allowing the described interactions to be presented in a more general and comprehensive way. In Figures 5–7 various graphs are presented for various combinations of process parameters for the bead-free fiber diameter (*D*). All the graphs are drafted using color scales which indicate how the values of the fiber diameter change with the modification of the studied factors. In addition, projections of the graphs on the *x*–*y* plane were made to make the data easier to analyze.

On the basis of this type of graph, it is possible to determine the conditions under which fibers with the expected size can be obtained. For example, following the graphs shown in Figure 5, the parameter range in which fiber diameters of about 1.65 μm (yellow stripes) are obtained can be read. For a minimum flow rate (*Q* = −1, Figure 5A), the electrical voltage should be varied in the range from *U* = 0 to *U* = 0.5 while the dynamic viscosity may be of any value. However, for the maximum flow rate (*Q* = +1, Figure 5B), the voltage range variation depends on the viscosity value – for example, for μ = 1 the voltage should be between *U* = 0.5 and *U* = 0.75. The actual values of the parameters can be obtained on the basis of an appropriate interpretation of extreme parameters, as it assumes a linear relationship between the analyzed factors and their high and low levels. For example, in the case of an electrical voltage, the value *U* = −1 corresponds to the actual value (*U*_r_): *U*_r_ = 7.5 kV and *U* = +1 is assigned to *U*_r_ = 15 kV. The next step is to determine the linear function *U*_r_ = *f*(*U*) analytically or graphically using calculation software. Afterwards, it can be easily obtained that for *U* = 0 there is *U*_r_ = 11.25 kV, for *U* = 0.5 the actual voltage is *U*_r_ = 13.13 kV, and for *U* = 0.75 we have *U*_r_ = 14.06 kV. In the same way all presented diagrams may be used.

According to Figure 5 which presents the simultaneous influence of the electrical voltage (*U*) and the dynamic viscosity of the polymer solution (µ) on the bead-free fiber diameter (*D*) at constant maximum (*Q* = 1) or minimum (*Q* = −1) flow rate, it is clear that the electrical voltage plays the predominant role in the process. When the flow rate is at the high level (Figure 5B), the impact of the dynamic viscosity (and thus the concentration) of the solution increases with decreasing electrical voltage. For the minimum flow rate (*Q* = −1), the dynamic viscosity impact on *D* is low, regardless of the electrical voltage level (Figure 5A). In both cases voltage enhancement results in a reduction of the fiber diameter (a change in *D* of around 1 µm). Interestingly, Tan et al. [1] demonstrated that the voltage had no significant effect on the size of the fibers. In this work, the diameter of the fibers changed little when the applied voltage was varied and for low concentrations of the polymer solution – practically not at all. However, the range of polylactide solution concentration in this study was between 1.25–4%, which is probably too narrow to obtain reliable results for general conclusions.

Regarding viscosity, while its value remains constant (Figure 6), the electrical voltage impact on the fiber diameter is larger than the impact of the flow rate, especially in the case of maximum viscosity (µ = 1, Figure 6B) when the fiber diameter is around 1 µm. The lower the electrical voltage, the thicker fibers are obtained. Shifting the flow rate from a low to high level causes a change in *D* of around 200–300 nm. Such an increase was also observed in the work by Pillay et al. [28].

Another relationship can be seen in Figure 7 comparing the effect of dynamic viscosity (µ) and flow rate (*Q*) on fiber diameter at constant electrical voltage (*U* = 1; *U* = −1). In both cases the viscosity has a considerably greater impact on fiber diameter than the flow rate. What is interesting is that for an electrical voltage at the high level (Figure 7B) the impact of the flow rate on fiber size depends on the value of the viscosity. When the dynamic viscosity level is high (µ > 0), the relationship between *D* and *Q* is directly proportional; in the opposite case (µ < 0) it is inversely proportional. In this case there is a specific range of factors (represented by the light-orange area on the graph in the Figure 7B) where slight changes in their value do not cause changes in the fiber diameter. This area can be treated as an area of safe and stable work.

#### Beaded fibers

An analogous factorial design was performed to examine the influence of the same factors as presented in the previous section on the bead size and the fiber size for bead-on-string mat structures. The high and low levels of selected process factors were reselected to values that allow beaded fibers to be obtained. The values are summarized in Table 3.

**Table 8:** Experimental variants for a 2^3^ factorial design including the average diameter of obtained beads (*d*_b_) and average diameter of beaded fibers (*d*). SD_b_ is the standard deviation of *d* and SD_db_ is the standard deviation of *d*_b_.

No.	*C* [%]	µ [Pa·s]	*Q* [mL/h]	*U* [kV]	*d* [µm]	SD_b_ [µm]	*d*_b_ [µm]	SD_db_ [µm]

1	8	0.043	0.6	7.5	0.271	0.120	5.67	1.38
2	8	0.043	0.6	15.0	0.226	0.088	3.08	0.74
3	8	0.043	1.2	7.5	0.272	0.130	4.31	1.29
4	8	0.043	1.2	15.0	0.282	0.085	5.08	1.56
5	12	0.107	0.6	7.5	0.687	0.175	9.24	2.09
6	12	0.107	0.6	15.0	0.544	0.080	5.88	1.06
7	12	0.107	1.2	7.5	0.816	0.182	12.81	2.61
8	12	0.107	1.2	15.0	0.705	0.168	8.19	1.55

According to the procedure outlined previously the values of coefficients were determined using Equation 6. The results of the calculations are listed in the Table 9.

**Table 9:** The model coefficients determining the impact of solution dynamic viscosity (µ), solution flow rate (*Q*) and electrical voltage (*U*) on the average beaded fiber diameter (*d*).

*b*_0_	*b*_μ_	*b_Q_*	*b_U_*	*b*_μ_*_Q_*	*b*_μ_*_U_*	*b_QU_*	*b*_μ_*_QU_*

0.475	0.213	0.043	−0.036	0.029	−0.027	0.011	−0.003

The corresponding model equation for the beaded fiber diameter is presented as:

[9]
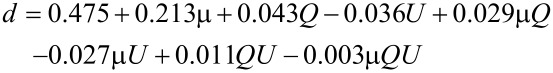


**Table 10:** The model coefficients determining the impact of solution dynamic viscosity (µ), solution flow rate (*Q*) and electrical voltage (*U*) on the average bead size (*d*_b_).

*c*_0_	*c*_μ_	*c_Q_*	*c_U_*	*c*_μ_*_Q_*	*c*_μ_*_U_*	*c_QU_*	*c*_μ_*_QU_*

6.783	2.248	0.815	−1.225	0.655	−0.770	0.263	−0.578

The corresponding model equation for the bead size is presented as:

[10]
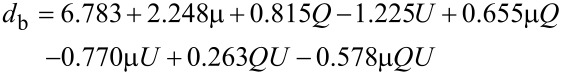


On the basis of the obtained model coefficients presented in Table 9 and Table 10, the dynamic viscosity of the polymer solution (µ) can be considered to be the parameter with the greatest influence on the average size of both beaded fibers and the beads. Since the coefficients *b*_µ_ and *c*_µ_ are positive, these relationships are directly proportional. For the beaded fiber diameter, the influence of the solution flow rate (*Q*) and electrical voltage (*U*) are comparable and much smaller than the impact of the viscosity. For the bead size, the values of coefficients *c*_µ_ and *c_U_* are an order of magnitude greater than the values of the other parameters. This suggests that µ and *U* are the most important factors in the process and that the factor *Q* plays a minor role in the process.

**Figure 8:**
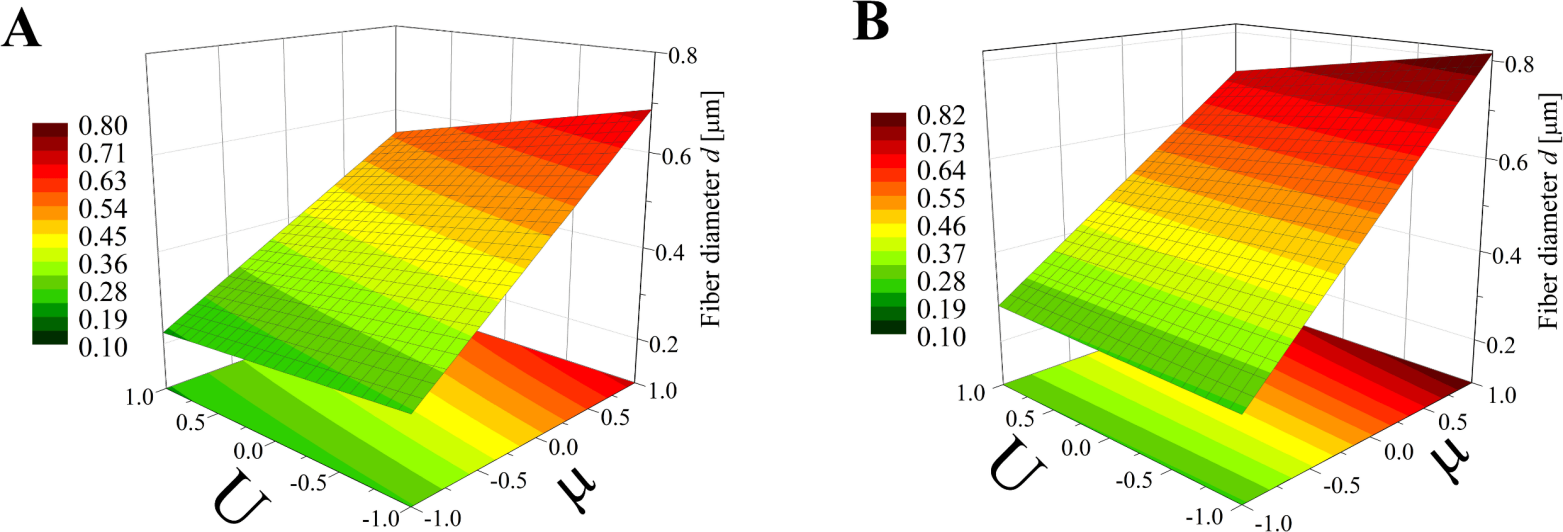
Response surface plots presenting the dependence of the diameter of the obtained beaded fibers (*d*) on the electrical voltage (*U*) and the dynamic viscosity (µ) of the polymer solution for (A) minimum flow rate (*Q* = −1) and (B) maximum flow rate (*Q* = +1)*.*

**Figure 9:**
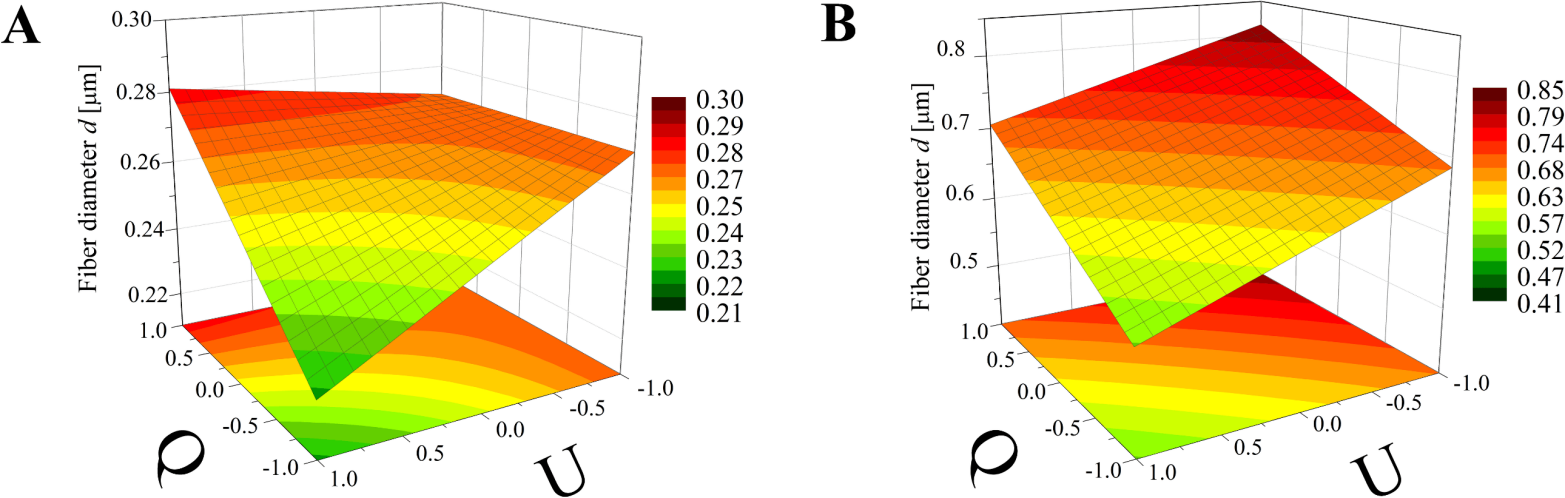
Response surface plots presenting the dependence of the diameter of the obtained beaded fibers (*d*) on the electrical voltage (*U*) and the flow rate (*Q*) of the polymer solution for (A) minimum dynamic viscosity (µ = −1) and (B) maximum dynamic viscosity (µ = +1).

**Figure 10:**
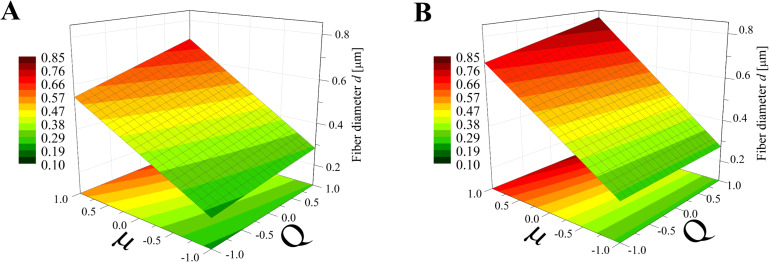
Response surface plots presenting the dependence of the diameter of the obtained beaded fibers (*d*) on the dynamic viscosity (µ) and the flow rate (*Q*) of the polymer solution for (A) maximum electrical voltage (*U* = +1) and (B) minimum electrical voltage (*U* = −1)*.*

**Figure 11:**
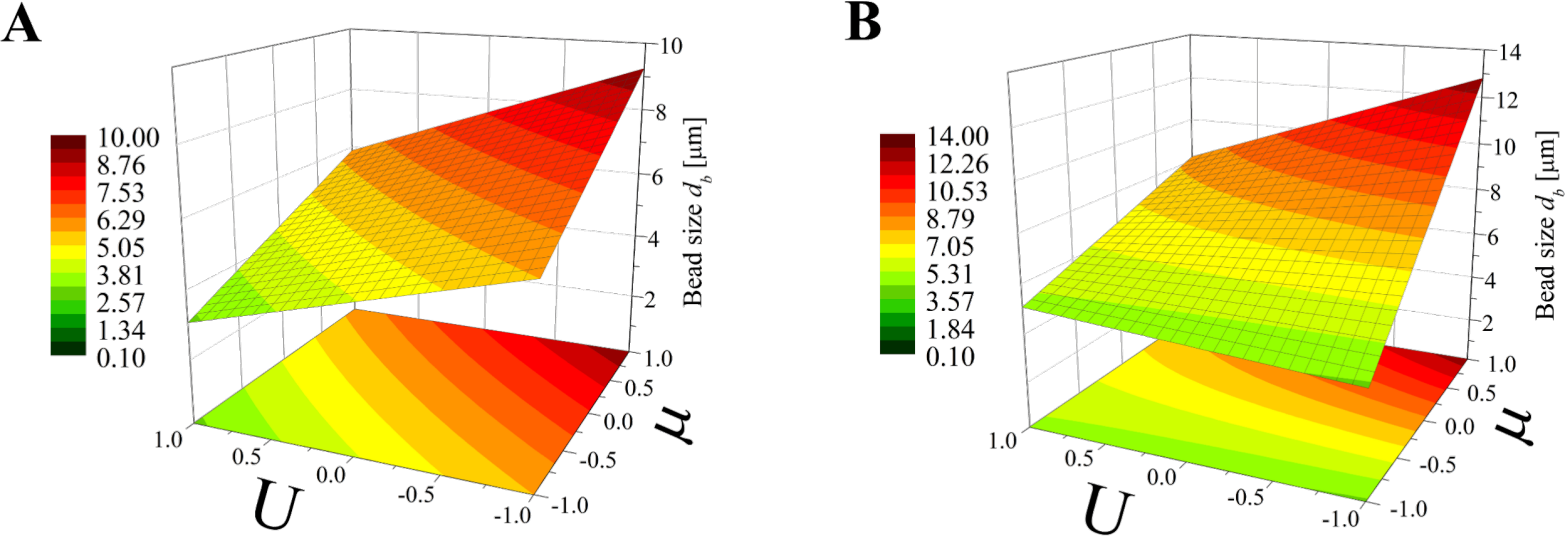
Response surface plots presenting the dependence of the size of obtained beads (*d*_b_) on the electrical voltage (*U*) and the dynamic viscosity (µ) of the polymer solution for (A) minimum flow rate (*Q* = −1) and (B) maximum flow rate (*Q* = +1).

**Figure 12:**
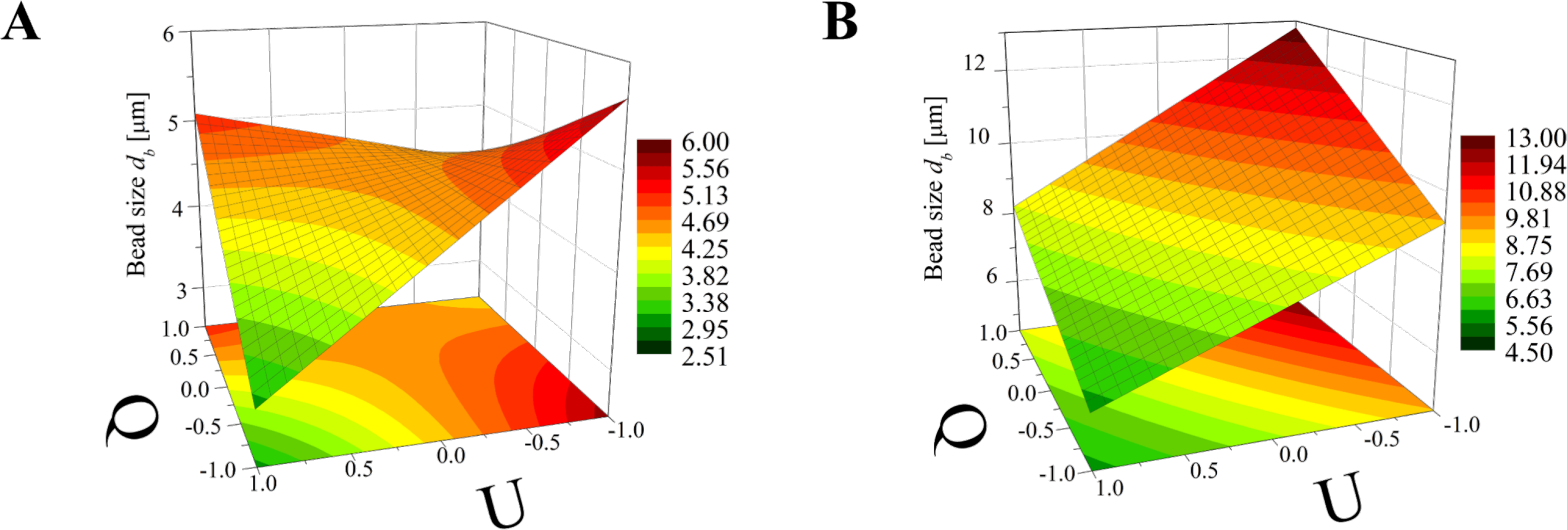
Response surface plots presenting the dependence of the size of obtained beads (*d*_b_) on the electrical voltage (*U*) and the flow rate (*Q*) of the polymer solution for (A) minimum dynamic viscosity (µ = −1) and (B) maximum dynamic viscosity (µ = +1)*.*

**Figure 13:**
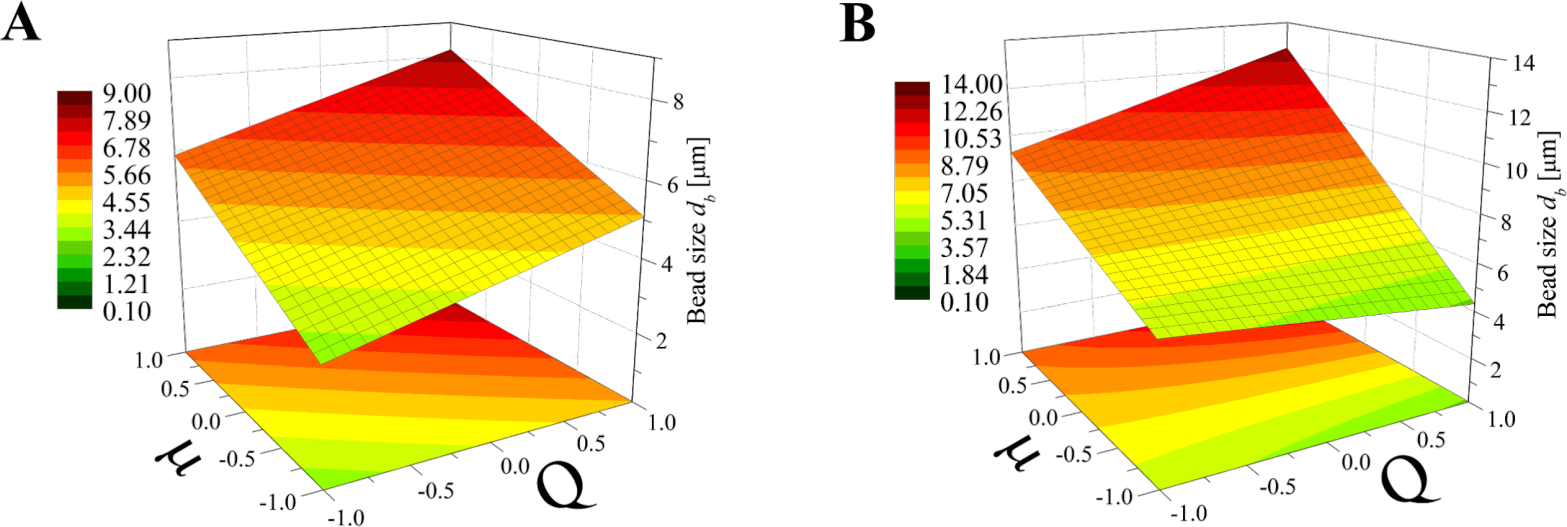
Response surface plots presenting the dependence of the size of obtained beads (*d*_b_) on the dynamic viscosity (µ) and the flow rate (*Q*) of the polymer solution for (A) minimum electrical voltage (*U* = −1) and (B) maximum electrical voltage (*U* = 1)*.*

Again, the obtained models have been used to create three-dimensional plots. Figures 8–10 and Figures 11–13 show graphs for various combinations of process parameters for the beaded fiber diameter *d* as well as the bead diameter *d*_b_, respectively. As previously, all the graphs have been drafted using color scales together with their projections on the *x*–*y* plane.

Comparing the graphs in Figures 8–10 and Figures 5–7, it can be stated that the fiber diameter of bead-on-string structures is 1–2 μm smaller than the bead-free fiber diameter, which seems to be obvious due to the fact that some part of the polymer was used to form beads. To the authors’ knowledge, the dependences between the diameter of bead-free and beaded fibers have not been studied yet.

The influence of the electrical voltage (*U*) and the dynamic viscosity of the polymer solution (µ) on the beaded fiber diameter (*d*) at constant maximum (*Q* = +1) or minimum (*Q* = −1) flow rate (Figure 8) is completely different than for the bead-free fiber diameter (*D*) (Figure 5). The solution dynamic viscosity has the largest impact on fiber size and causes a change of around 700 nm when its value increases from the low level (µ = −1) to the high level (µ = 1)*.* In this case, the electrical voltage impact is about tenfold weaker.

Comparing Figure 6 with Figure 9, which corresponds to the case of constant viscosity (µ = 1, µ = −1), another change in the effect of process parameters on the fiber size can be noticed. The flow rate has a much larger influence on the beaded fiber diameter (Figure 9) than in the case of bead-free fibers (Figure 6) and is quantitatively comparable to the effect of the electrical voltage. However, the relationship between *d* and *Q* is directly proportional, while the relationship between *d* and *U* is inversely proportional.

In the case of a constant electrical voltage value (*U* = 1, *U* = −1) (Figure 10), the viscosity has a much greater effect on the beaded fiber diameter than the flow rate, similar to the case of bead-free fibers (Figure 7). However, differently to them, it is always a directly proportional relationship. Regardless of the value of the viscosity or voltage, the flow has little effect on the diameter, causing it to change up to 0.1 μm, whereas when the viscosity changes from a low level to high level, an increase in diameter of up to 0.7 µm can be observed (Figure 10).

Concerning the beads, the dependencies studied in the work are similar to those of beaded fibers, although there are some differences. On the basis of comparison of the graphs in Figures 8–10 and Figures 11–13 it can be established that the bead diameter is about ten times bigger than the diameter of the beaded fiber (Figures 8–10) where they are located. Fong et al. [17] studied beaded PEO nanofibers formed during electrospinning and a similar relationship between the size of the fibers and beads were determined by these authors.

According to Figure 11 the dynamic viscosity of the solution and the electrical voltage have a similar influence on the bead size; however, neither of these values has a greater impact as that observed for the case of the bead-free (Figure 5) or beaded fiber (Figure 8) diameter. Depending on the *Q* value, the bead diameter increases from about 6 µm to 9 µm (*Q* = −1) or from 4 µm to 13 µm (*Q* = 1) with a change of dynamic viscosity from a low to high level. In contrast, the relationship between *d*_b_ and *U* is inversely proportional. Such dependencies were also previously observed by Li et al. [21] and Fong et al. [17].

The simultaneous effect of the flow rate and electrical voltage on the bead size at the constant maximum (μ = 1) or the minimum (μ = −1) solution viscosity is presented in Figure 12. It shows that for low μ values (Figure 12A) the bead size *d*_b_ increases with increasing *Q* when the *U* level is high (*U* > 0) and decreases in the opposite case (*U* > 0)*.* For all values of μ (Figure 12B) the relationship between *d*_b_ and *Q* is always directly proportional and the relationship between *d*_b_ and *U* is inversely proportional.

Analyzing Figure 13, it can be concluded that for a constant *U* level, the effects of μ and *Q* on the bead size *d*_b_ is comparable for the low voltage level (*U* = −1, Figure 13A) with the fact that for larger viscosities and flow rates, larger beads are obtained. When the *U* level is high (*U* = 1, Figure 13B) the solution viscosity changes have a dominant effect on the bead size, causing them to increase from 4 µm up to 13 µm.

## Conclusion

This work describes the study of the influence of selected factors (flow rate of poly(vinyl pirrolidone) solution, polymer solution viscosity and applied electrical voltage) on the average diameter of fibers and beads that are produced by the electrospinning process. A full 2^3^ factorial design was performed in order to establish the abovementioned relationships. Although only four solutions were used in the study and just sixteen experiments were carried out, fiber diameters and bead sizes were obtained for all values between high and low levels of factors. The results of this factorial design establish the conditions for the production of fibers (with and without beads) and beads of a desired size in an electrospinning process using PVP/ethanol solution.

According to the results presented in this study, it can be stated that the influence of process parameters on the obtained fiber and bead sizes in the electrospinning process depends on which structure is considered. The bead-free fiber diameter depends mainly on the electrical voltage, while the viscosity and the flow rate are less important. On the other hand, the greatest influence on the diameter of beaded fibers and beads was observed for the dynamic viscosity of the feed solution, and the electrical voltage was the factor with a second major impact on the fiber and bead size. Given the significance of interactions between the studied factors they cannot be omitted during the analysis of the discussed process. This work proved that in order to obtain fibrous mats with expected characteristics features, there is no need to perform many laboratory tests, as the influence of process conditions on the properties of the final material can be determined using mathematical methods. The presented method can be used as a tool for process design and for identification of potentially optimal conditions for obtaining electrospun fibers with the desired features in a simpler, faster and less expensive manner. Given a mathematical description of the process, one can easily check its adequacy, optimize it and estimate which parameters play a predominant role. The approach described in this work can provide the basis for further research on the elaboration of optimal electrospinning process conditions.
